# Unusual susceptibility to vancomycin in *Elizabethkingia meningoseptica*: mechanisms and clinical implications

**DOI:** 10.3389/fmicb.2026.1767980

**Published:** 2026-04-13

**Authors:** Qi Sun, Miao Sun, Jia Du, Heyuan Guan, Ze Li, Chun Yang, Baoyu Sun, Jiancheng Xu, Wei Xia

**Affiliations:** 1 College of Medical Technology, Beihua University, Jilin, China; 2 Department of Laboratory Medicine, The First Hospital of Jilin University, Changchun, China

**Keywords:** biofilms, *Elizabethkingia meningoseptica*, gram-negative bacteria, outer membrane permeability, vancomycin

## Abstract

*Elizabethkingia meningoseptica*, an environmental, non-fermenting Gram-negative bacillus, poses a serious clinical challenge due to its intrinsic multidrug resistance. Paradoxically, the pathogen exhibits exceptional *in vitro* susceptibility to vancomycin, a glycopeptide antibiotic normally directed only against Gram-positive organisms. This review systematically synthesizes recent evidence—published since earlier key reviews—on this paradoxical susceptibility. The review clarifies taxonomic evolution and identification challenges within the genus *Elizabethkingia*, highlighting how frequent misidentification between *E. meningoseptica* and *E. anophelis* has confounded historical susceptibility data. It proceeds to critically evaluate methodological variations in antimicrobial susceptibility testing and their impact on vancomycin susceptibility reporting. It discusses emerging mechanistic insights, including hypothesized increased outer-membrane permeability and biofilm inhibition at sub-minimum inhibitory concentrations, as plausible but not yet conclusively proven explanations for vancomycin activity. Finally, it proposes evidence-based treatment strategies, emphasizing susceptibility-guided combination regimens over vancomycin monotherapy. This updated synthesis aims to provide both theoretical grounding and pragmatic clinical guidance for managing multidrug-resistant *Elizabethkingia* infections.

## 1 Introduction

*Elizabethkingia meningoseptica* (*E. meningoseptica*) is an opportunistic pathogen of increasing clinical concern and an important cause of nosocomial infections, capable of causing severe diseases such as meningitis, sepsis and pneumonia, particularly in neonates and immunocompromised patients (Lin et al., 2019). Due to the presence of intrinsic Ambler class A extended-spectrum *β*-lactamases and class B metallo-*β*-lactamases, *E. meningoseptica* is resistant to most β-lactam antibiotics and exhibits reduced susceptibility to several other antimicrobial classes, making treatment particularly challenging (Zajmi et al., 2022). Vancomycin, a glycopeptide antibiotic that inhibits cell wall synthesis by binding to the D-Ala-D-Ala moiety of peptidoglycan precursors, is primarily active against Gram-positive bacteria (Jones, 2006; Stogios and Savchenko, 2020). While Gram-negative bacteria are generally considered intrinsically resistant to vancomycin because of the permeability barrier of the outer membrane, several studies have reported that *E. meningoseptica* may exhibit *in vitro* susceptibility to vancomycin, and clinical use has occasionally been associated with therapeutic benefit (Prajnha et al., 2024; Jean et al., 2017; Lin et al., 2023). However, the mechanisms underlying this apparent susceptibility remain unclear, and the absence of standardized susceptibility testing methods continues to generate debate regarding its clinical significance. A previous review by Jean et al. (2017) summarized earlier evidence and concluded that intravenous vancomycin monotherapy was not consistently effective.

To build upon this work, the present review summarizes studies published between January 2014 and December 2024. The aim is to evaluate the reported susceptibility of *Elizabethkingia* species to vancomycin, explore potential mechanisms, and discuss current controversies and future research directions. To provide a transparent overview of the literature considered, we conducted a targeted narrative review using PubMed, Scopus, and Web of Science. Search terms included “*Elizabethkingia meningoseptica*,” “*Elizabethkingia anophelis*,” “*Chryseobacterium meningosepticum*,” “*Flavobacterium meningosepticum*,” “vancomycin” and “susceptibility.” We prioritized published reports containing *in vitro* minimum inhibitory concentrations (MICs), antimicrobial susceptibility profiles, or clinical treatment outcomes involving *Elizabethkingia*. Studies were excluded if they lacked specific susceptibility data, focused exclusively on environmental isolates without clinical relevance, or if sufficient data could not be extracted to support a rigorous analysis.

## 2 Taxonomic evolution and identification challenges of *E. meningoseptica*

The taxonomic history of *E. meningoseptica* reflects the transition in bacterial classification from phenotypic characteristics to genomic analyses. In the 1950s, a non-fermentative Gram-negative rod was initially isolated from cases of neonatal meningitis by the U.S. Centers for Disease Control and Prevention (CDC) and temporarily designated as group IIa. In 1959, based on morphological and biochemical characteristics, it was formally classified as *Flavobacterium meningosepticum* within the genus *Flavobacterium* (King, 1959). With advances in molecular biology, genetic and biochemical evidence showed that this organism formed a distinct rRNA cluster closely related to core species of the genus *Chryseobacterium*, leading to its reclassification in 1994 as *Chryseobacterium meningosepticum* (Vandamme et al., 1994). In 2005, through polyphasic taxonomic approaches including 16S rRNA gene phylogeny and DNA–DNA hybridization, Kim et al. (2005) revealed that *C. meningosepticum* and *C. miricola* formed a monophyletic lineage separate from the genus *Chryseobacterium*. Consequently, a new genus, *Elizabethkingia*, was proposed in honor of Elizabeth O. King, who first identified the organism, with the two species renamed as *E. meningoseptica* and *E. miricola*, respectively. Recent in-depth studies based on polyphasic taxonomy and metagenomic phylogeny have expanded the genus *Elizabethkingia* to seven valid species: *E. meningoseptica*, *E. anophelis*, *E. miricola*, *E. bruuniana*, *E. occulta*, *E. ursingii*, and *E. argenteiflava* (Lin et al., 2022).

*E. meningoseptica* and its closely related species *E. anophelis* are important pathogens in healthcare-associated infections, sharing 16S rRNA gene sequence similarity as high as 98.82% (Lin et al., 2023). Phenotypic testing cannot reliably distinguish between them. Even when using matrix-assisted laser desorption/ionization time-of-flight mass spectrometry (MALDI-TOF MS), misidentification as *E. meningoseptica* frequently occurs if reference spectra for *E. anophelis* are absent in the database (Lin et al., 2019; Takei et al., 2025). Although whole-genome sequencing represents the most accurate method for elucidating the genetic diversity within the genus *Elizabethkingia* and for species identification, its high cost and procedural complexity hinder its routine use in clinical laboratories (Zajmi et al., 2022; Takei et al., 2025). A study from Singapore examined 79 bloodstream isolates; initial MALDI-TOF MS identified 96.2% as *E. meningoseptica* and 3.8% as *E. miricola*, yet 16S rRNA sequencing reclassified 98.7% of them as *E. anophelis* (Chew et al., 2018). This finding is consistent with research from Chinese Taiwan, which retrospectively reviewed clinical microbiology laboratory data and found that a significant number of clinical isolates initially reported as *E. meningoseptica* were ultimately confirmed to be *E. anophelis* (Lin et al., 2018). Such misidentification has confounded early antimicrobial susceptibility data, suggesting that many infections successfully treated with vancomycin and historically attributed to *E. meningoseptica* may actually have been caused by *E. anophelis*. Accurate species identification is crucial for clarifying epidemiological characteristics, resistance mechanisms, and clinical management strategies. *E. anophelis* is a predominant pathogen in healthcare settings in Southeast Asia, whereas *E. meningoseptica* is more commonly reported in other regions (Zajmi et al., 2022). Furthermore, *E. miricola* demonstrates higher susceptibility to fluoroquinolones, while both *E. anophelis* and *E. meningoseptica* often show greater susceptibility to piperacillin-tazobactam (Comba et al., 2022; Han et al., 2017). Therefore, it is imperative to integrate reliable molecular techniques, such as 16S rRNA or *rpoB* gene sequencing, into routine practice to reduce misdiagnosis and optimize antimicrobial therapy.

## 3 Methodological controversies in *in vitro* susceptibility testing

The reported *in vitro* susceptibility of *Elizabethkingia meningoseptica* to vancomycin shows substantial interstudy variability (The data are shown in Table 1), largely attributable to the absence of standardized antimicrobial susceptibility testing (AST) protocols and genus-specific interpretive criteria. To date, no internationally recognized breakpoints have been established for *Elizabethkingia* species. Consequently, broth microdilution (BMD) is widely recommended as the reference method for the determination of reliable MIC values (Lin et al., 2023; Kuo et al., 2021). In the absence of dedicated guidelines, most studies report susceptibility results according to the Clinical and Laboratory Standards Institute (CLSI) M100 criteria for non-Enterobacterales (CLSI, 2024). For vancomycin, interpretive criteria are frequently extrapolated from *Enterococcus* spp., defining susceptibility as an MIC ≤ 4 μg/mL or a disk diffusion inhibition zone ≥17 mm using a 30 μg disk (CLSI, 2024). However, accumulating evidence indicates that these surrogate breakpoints have limited applicability to *Elizabethkingia* species and may lead to misinterpretation of antimicrobial activity and potential clinical efficacy (Jean et al., 2017).

**Table 1:** Comparison of susceptibility testing methods for *Elizabethkingia meningoseptica*.

Testing method	Advantages	Limitations	Applicability
Disk diffusion	Simple operation; Low cost	Unreliable results; False susceptibility	Not recommended
Broth/Agar microdilution	Accurate results; Gold standard	Technically complex; Time-consuming	Recommended as reference method
E-test	Simple operation; Provides MIC	Results require confirmation	Useful alternative
Automated susceptibility systems	High throughput; Automated	Limited database accuracy	Requires result verification

Beyond interpretive criteria, variability among testing methodologies further complicates the assessment. Comparative studies have demonstrated poor concordance between reference methods and alternative testing approaches, including E-test and disk diffusion (Huang, 2024). Among these, disk diffusion is particularly prone to overestimating vancomycin susceptibility and is therefore not recommended for routine testing of *Elizabethkingia* (Fraser and Jorgensen, 1997). Automated AST systems, such as VITEK 2, also exhibit methodological limitations, potentially due to insufficient database calibration for rare non-fermenting bacilli. Previous reports indicate that VITEK 2 misclassified vancomycin susceptibility in approximately 3.7% of isolates, with categorical discrepancy rates exceeding 1.5% for agents including ciprofloxacin, moxifloxacin, and vancomycin (Kuo et al., 2021). In addition, interlaboratory differences in experimental parameters—such as culture media composition, inoculum density, and incubation duration—may further influence results. Importantly, most studies do not report method-specific error rates or pharmacokinetic/pharmacodynamic correlations, thereby limiting the clinical interpretability of susceptibility categories. Consequently, the establishment of standardized AST methodologies and validated species-specific interpretive breakpoints is essential to reduce methodological bias, clarify current controversies, and improve the reliability of clinical decision-making.

## 4 Potential mechanisms underlying vancomycin susceptibility in *Elizabethkingia*

Given the methodological uncertainty discussed above, it remains necessary to determine whether the reported vancomycin susceptibility of *Elizabethkingia meningoseptica* reflects a genuine biological phenotype or experimental artifact. In this context, several mechanistic hypotheses have been proposed. Importantly, however, the strength of evidence supporting these mechanisms varies considerably, and a clear distinction between experimentally validated findings and speculative inference is essential.

### 4.1 Outer membrane permeability: evidence and species-specific considerations

**Figure 1:**
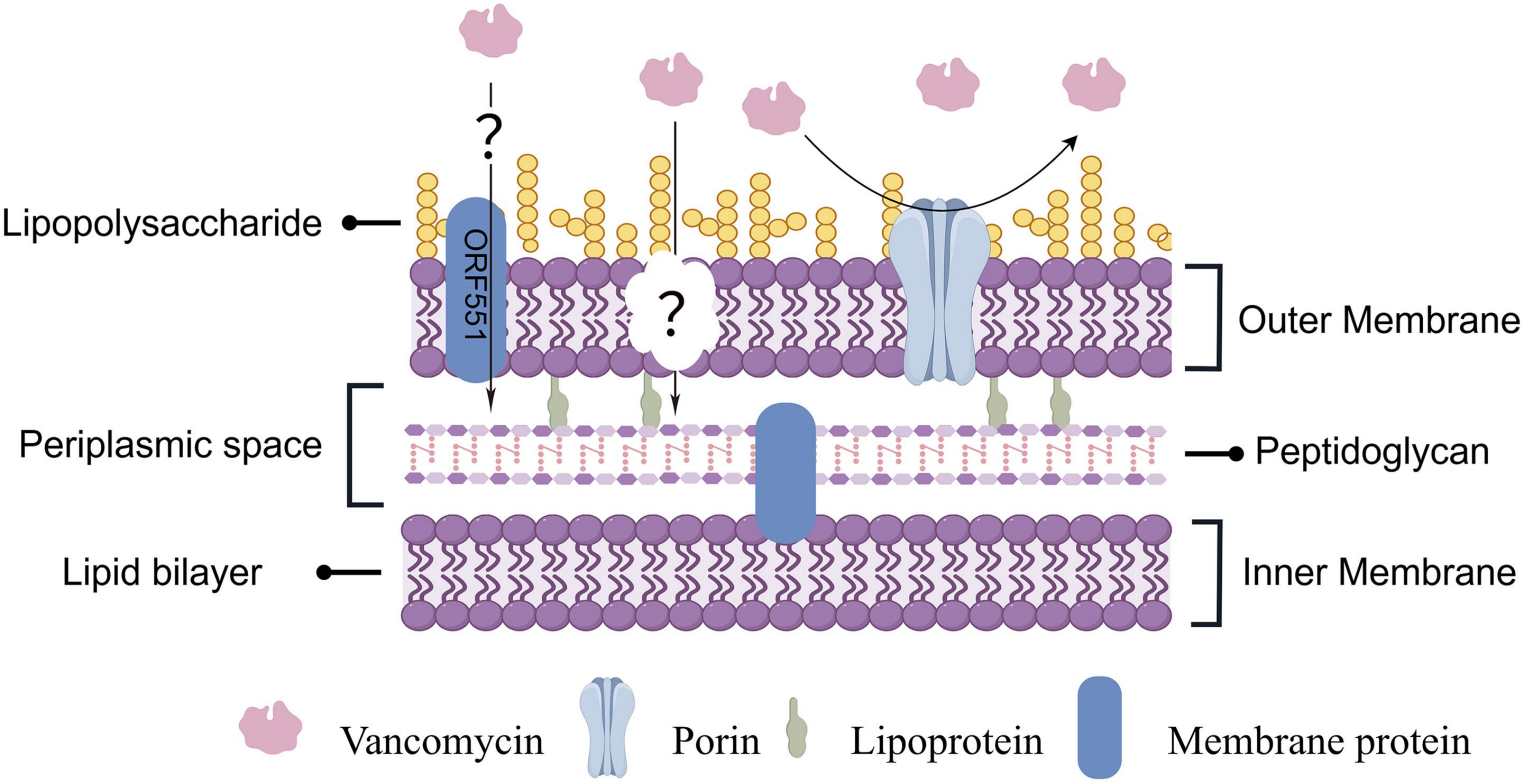
Schematic diagram of the cell wall structure of a gram-negative bacterium.

It is well established that intrinsic glycopeptide resistance in Gram-negative bacteria primarily results from exclusion by the outer membrane (This case is depicted in Figure 1). Experimental models in *Escherichia coli* have demonstrated that mutations affecting lipopolysaccharide (LPS) assembly or outer membrane protein composition can increase permeability to high-molecular-weight agents, including vancomycin (Huang et al., 2008). Similarly, polymyxin-mediated disruption of LPS enhances penetration of otherwise excluded antibiotics (Vaara, 2019), and chemical modification of vancomycin has been shown to facilitate outer membrane transit (Antonoplis et al., 2019). These studies demonstrate that altered envelope permeability is, in principle, sufficient to permit glycopeptide activity in engineered or chemically perturbed Gram-negative organisms. However, direct evidence for increased outer membrane permeability in wild-type *E. meningoseptica* remains limited. To date, no quantitative permeability assays, fluorescent vancomycin uptake studies, or outer membrane proteomic analyses have conclusively demonstrated enhanced glycopeptide penetration in susceptible isolates. Therefore, although altered permeability is biologically plausible, it remains unproven in this species.

Recent genomic data provide indirect support for envelope-associated mechanisms. In *Elizabethkingia anophelis*, vancomycin-resistant (16 μg/mL) mutants selected under antibiotic pressure were found to harbor mutations in the *vsr1-ORF551* operon (Johnson et al., 2024). The vsr1 gene encodes a PadR-family transcriptional regulator implicated in membrane stress responses. Disruption of this regulatory pathway was associated with elevated vancomycin MICs and morphological changes consistent with envelope instability. Although these findings suggest that membrane homeostasis may influence glycopeptide susceptibility, extrapolation to *E. meningoseptica* requires caution, as functional validation in this species has not yet been performed. Collectively, current evidence supports the conceptual feasibility of permeability-mediated susceptibility but does not yet establish it as a confirmed mechanism in *E. meningoseptica*.

### 4.2 Biofilm modulation: a contributing factor, not a direct mechanism

Biofilms are structured communities of bacterial cells adhered to biotic or abiotic surfaces, encased within a self-secreted polymeric matrix composed of extracellular polysaccharides, proteins, and DNA (Shree et al., 2023). As biofilms enhance bacterial environmental persistence, reduce host immune recognition, and contribute significantly to antibiotic resistance, various strategies have been developed to prevent and eradicate biofilms (Kalia et al., 2023). One such approach involves the use of antibiotics at sub-inhibitory concentrations. For instance, vancomycin administered at sub-MIC levels has been shown to inhibit biofilm formation and significantly reduce adhesion in enterococci (Hashem et al., 2017).

Genomic analyses indicate that *E. meningoseptica* possesses genes associated with *curli* fiber biosynthesis, hemagglutinins, and capsular polysaccharide synthesis—all of which are crucial molecular determinants for biofilm formation and adhesion (Chen et al., 2017; Chen et al., 2019). Compared with *E. anophelis* and *E. miricola*, *E. meningoseptica* exhibits significantly greater biofilm-forming capacity, and the underlying genetic determinants are highly conserved. Biofilm formation not only enhances its persistence on medical devices and in water systems but also markedly increases the difficulty of infection control (Holcomb et al., 2024).

Therefore, the inhibition of biofilm formation may represent another important mechanism by which vancomycin may act effectively against *Elizabethkingia*. Studies have shown that vancomycin at sub-MIC [4 μg/mL (0.25 × MIC)] can effectively suppress biofilm formation and host cell adhesion in *Elizabethkingia* (Yang et al., 2024), which is particularly relevant for controlling persistent infections in hospital environments. Nevertheless, biofilm suppression should not be equated with bactericidal susceptibility. Sub-MIC effects may reflect modulation of regulatory pathways involved in surface attachment rather than effective access to peptidoglycan targets. Moreover, the concentrations required for biofilm inhibition may not correspond to clinically achievable serum levels. Therefore, although biofilm modulation may contribute to observed *in vitro* synergy or clinical combination efficacy, it is unlikely to represent a primary mechanistic basis for MIC-defined susceptibility. Instead, it may function as an adjunctive phenomenon that modifies phenotypic tolerance rather than intrinsic glycopeptide entry or target engagement.

### 4.3 Genomic determinants: lack of classical resistance genes and alternative pathways

Unlike classical vancomycin-resistant Gram-positive organisms, Elizabethkingia lacks acquired van gene operons that mediate D-Ala-D-Lac substitution of the peptidoglycan precursor (Stogios and Savchenko, 2020). This absence suggests that susceptibility is unlikely to be governed by canonical target modification mechanisms. Instead, variability in outer membrane proteins, efflux pump repertoires, and envelope stress response regulators identified in comparative genomic analyses (Chen et al., 2017; Chen et al., 2019) may modulate antibiotic entry or retention.

Importantly, no study has yet demonstrated a direct causal link between specific genetic determinants and vancomycin susceptibility in *E. meningoseptica*. Thus, the molecular basis of the reported phenotype remains undefined. In addition to intrinsic genomic factors, some studies have proposed context-dependent effects. For example, while controlling Gram-positive cocci in mixed infections, vancomycin may also partially disrupt the cell wall of *Elizabethkingia*, thereby exerting synergistic effects with other antimicrobial agents (Seong et al., 2020). However, such observations likely reflect indirect or ecological interactions rather than primary molecular susceptibility mechanisms.

Transcriptomic profiling under vancomycin exposure, CRISPR-based gene disruption in clinical isolates, and quantitative permeability assays using fluorescent vancomycin analogs will be required to establish mechanistic causality.

Taken together, current mechanistic explanations remain biologically plausible but incompletely validated. The available evidence supports the hypothesis that envelope-associated or context-dependent factors may modulate vancomycin activity; however, definitive experimental confirmation in *E. meningoseptica* is still lacking. This distinction between plausibility and proof is critical for avoiding overinterpretation of the paradoxical susceptibility phenotype.

## 5 Resistance mechanisms and clinical treatment strategies for *E. meningoseptica*

*E. meningoseptica* is a significant opportunistic nosocomial pathogen. Its multidrug resistance, mediated by diverse genetic determinants and molecular pathways, frequently renders conventional therapeutic regimens ineffective. Consequently, the development of novel and efficacious anti-infective strategies represents a central research priority.

Available studies suggest that minocycline is the most active antibiotic against *E. meningoseptica*, with susceptibility rates reaching up to 91.3% (Lin et al., 2018). Additionally, piperacillin/tazobactam, trimethoprim/sulfamethoxazole (TMP-SMX), and fluoroquinolones (such as ciprofloxacin and levofloxacin) have also demonstrated considerable antimicrobial activity (Huang et al., 2018; Ma et al., 2023). One study reported susceptibility rates of 96.0% for minocycline and 77.0% for TMP-SMX, whereas tobramycin and colistin were largely ineffective (Alhuthil et al., 2024). Therefore, the former agents are recommended as first-line therapeutic options for infections caused by *E. meningoseptica* (The data are shown in Table 2).

**Table 2:** Recommended anti-infective regimens for *Elizabethkingia meningoseptica*.

Species	Preferred agent(s)	Alternative agent(s)	Notes	Guidelines
*Elizabethkingia meningoseptica*	Piperacillin-tazobactam, Ciprofloxacin, TMP-SMX	Levofloxacin, Rifampin, Linezolid, Minocycline	Resistant to penicillins, cephalosporins, carbapenems, aminoglycosides, and polymyxins. The efficacy of vancomycin remains controversial and should be used with caution.	National Guidelines for Antimicrobial Therapy (3rd Edition) (National Expert Committee on Rational Drug Use, 2023)
	Levofloxacin or TMP-SMX	Ciprofloxacin, Minocycline	Resistant to penicillins, cephalosporins, carbapenems, aminoglycosides, and vancomycin.	The Sanford Guide to Antimicrobial Therapy (50th Edition) (Gilbert et al., 2020)

Several case reports indicate that combination regimens including vancomycin and other antibiotics yield favorable outcomes, particularly in the treatment of meningitis and bacteremia (Prajnha et al., 2024; Hashmi et al., 2023). *In vitro* and clinical evidence support the use of vancomycin in combination with ciprofloxacin, rifampin, or piperacillin/tazobactam. However, monotherapy with vancomycin is not recommended due to its uncertain efficacy (Jean et al., 2017; Jean et al., 2014).

It is noteworthy that despite apparent *in vitro* susceptibility to certain antibiotics, clinical treatment failures may still occur, suggesting the presence of adaptive resistance mechanisms (Prajnha et al., 2024; Stewart et al., 2023). Thus, in clinical practice, it is recommended to implement personalized treatment based on AST results. Close monitoring of the patient’s response is essential, and combination therapy regimens should be considered when necessary to overcome potential adaptive resistance and improve treatment success rates.

## 6 Discussion and conclusion

The clinical management of *E. meningoseptica* infections remains challenging due to its multidrug-resistant profile. While *in vitro* susceptibility data support the use of core agents such as minocycline, piperacillin-tazobactam, trimethoprim-sulfamethoxazole, and fluoroquinolones, discrepancies persist among international guidelines regarding first-line therapy and the role of vancomycin. These differences stem from historical species misidentification, methodological variability in AST, and geographical resistance patterns. As discussed, the interpretation of vancomycin susceptibility is obscured by taxonomic ambiguities and inconsistent AST methodologies. Misidentification of *E. anophelis* as *E. meningoseptica* has confounded historical data, while the absence of standardized breakpoints and variations in AST platforms complicate the interpretation of MIC results.

Importantly, recent literature suggests that the atypical vancomycin susceptibility profile is likely a conserved trait across the *Elizabethkingia* genus rather than being restricted to *E. meningoseptica* alone. Proposed mechanisms, such as altered outer membrane permeability and biofilm modulation, are biologically plausible. However, as noted in this review, many of these mechanistic insights are largely extrapolated from findings in *E. anophelis*. The utilization of *E. anophelis* as a proxy for *E. meningoseptica* in these studies further underscores the genus-wide nature of vancomycin activity. Nonetheless, these hypotheses require direct experimental validation through gene editing and proteomics specifically in *E. meningoseptica* to confirm species-specific molecular determinants.

Given these uncertainties, vancomycin should not be used as monotherapy. Instead, combination therapy, guided by reliable MIC data and standardized AST methods, should be prioritized. Treatment decisions should integrate local epidemiology, infection site, and patient-specific factors. Future research should focus on establishing standardized, species-specific AST methods, elucidating the molecular mechanisms of vancomycin susceptibility across the Elizabethkingia genus, and exploring novel therapeutic strategies, such as new antibiotics, phage therapy, and anti-biofilm agents, to address this growing clinical challenge.
